# Supplementary material for: Belief inference for hierarchical hidden states in spatial navigation
Source: Commun Biol. 2024 May 21;7:614. doi: 10.1038/s42003-024-06316-0 (PMC11109253; doi:10.1038/s42003-024-06316-0)
Supplement: Supplementary file 2 — Supplementary information [file 42003_2024_6316_MOESM2_ESM.pdf]

Supplementary information for "Belief inference for hierarchical hidden states in spatial navigation"

Risa Katayama[1,2]*, Ryo Shiraki[1], Shin Ishii[1,3,4], and Wako Yoshida[5,6]†

[1] Graduate School of Informatics, Kyoto University, Kyoto, 606-8501 Japan
[2] Department of AI-Brain Integration, Advanced Telecommunications Research Institute International, Kyoto, 619-0288 Japan
[3] Neural Information Analysis Laboratories, Advanced Telecommunications Research Institute International, Kyoto, 619-0288 Japan
[4] International Research Center for Neurointelligence, the University of Tokyo, Tokyo, 113-0033, Japan
[5] Department of Neural Computation for Decision-making, Advanced Telecommunications Research Institute International, Kyoto, 619-0288, Japan
[6] Nuffield Department of Clinical Neuroscience, University of Oxford, Oxford, OX3 9DU, UK

* Correspondence: katayama.risa.8d@kyoto-u.ac.jp
† Senior author

Supplementary Figures

Supplementary Fig. 1: Comparison of behavioural results between the behavioural and imaging experiments.

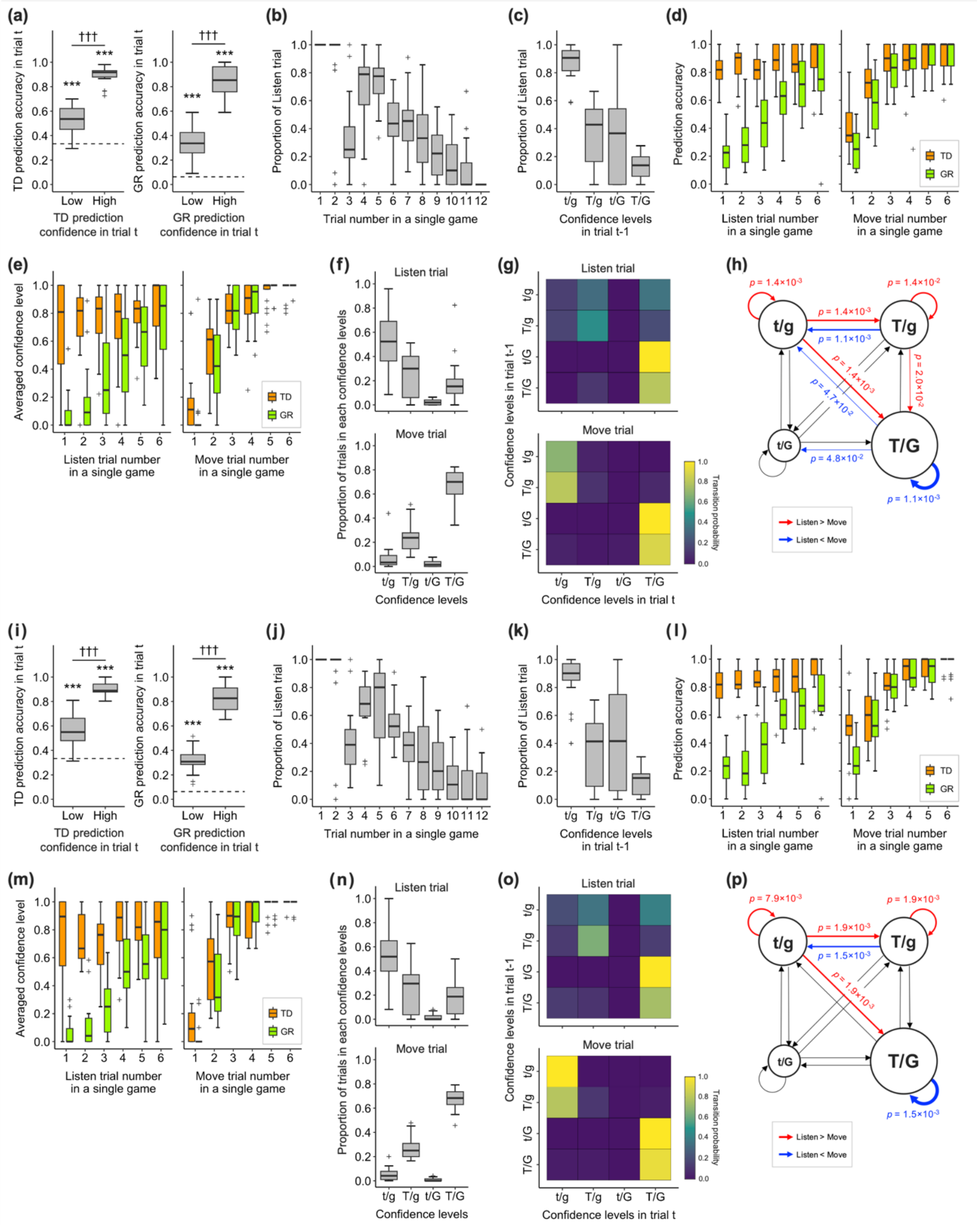

(a-h) and **(i-p)** show the behavioural results of analysing data from the behavioural (outside of the scanner) and imaging experiments, respectively. The analysis methods employed are the same as those shown in Fig. 2. The statistical results of the behavioural data did not change in

either experiment, confirming that the participants' performance did not vary across experimental settings.

Supplementary Fig. 2: Block diagram of the proposed hierarchical inference model (a) and those of the alternative models; the top-down inference model (b) and the parallel inference model (c).

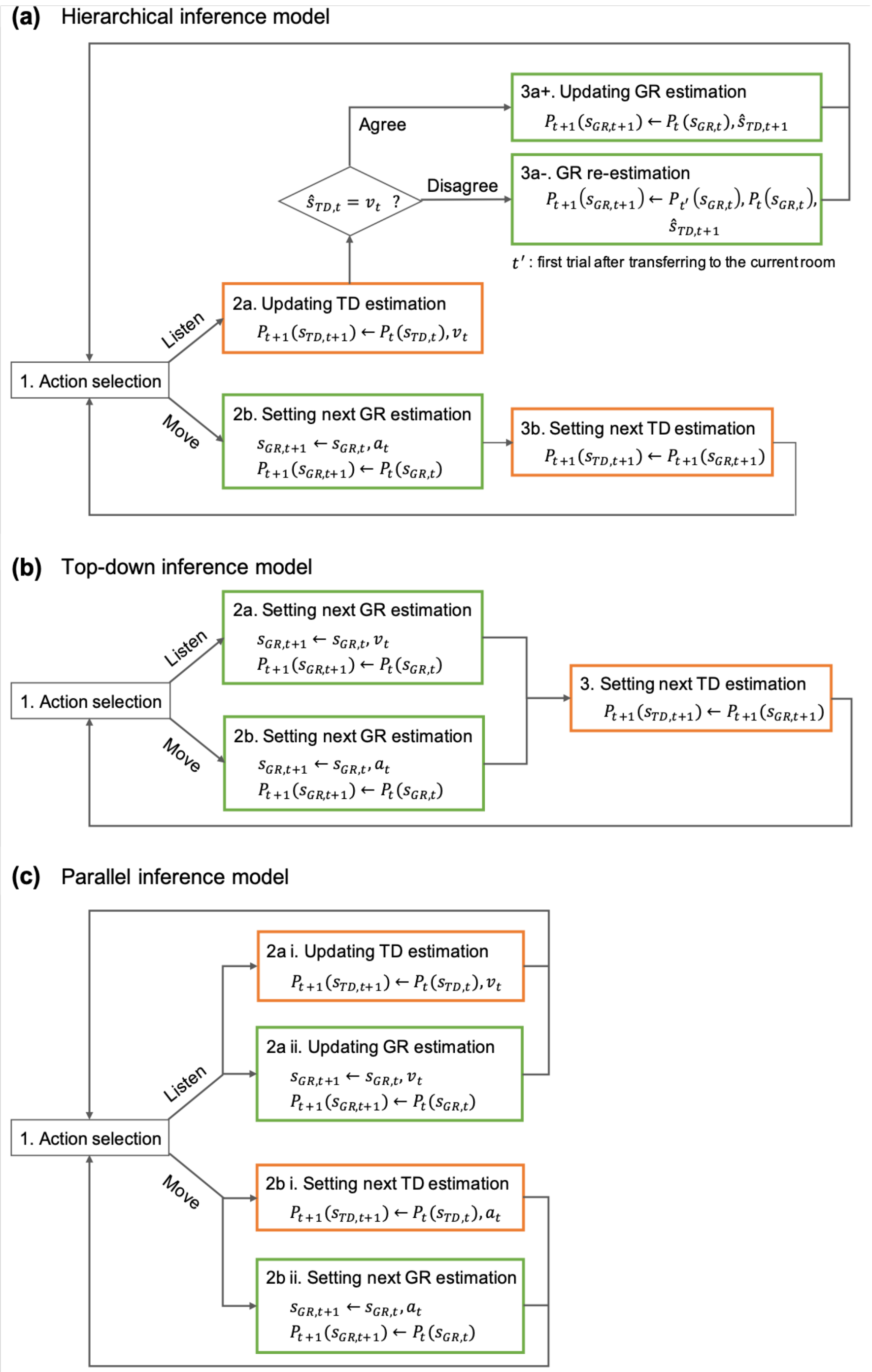

The hierarchical inference model infers the probability distributions of the upcoming tiger door

position ($s_{TD,t+1}$) and grid location ($s_{GR,t+1}$) based on the sequence of participant's actions (a_t) and observations of a tiger-roar (v_t). This model assumes that when the participants choose to listen, they infer the tiger door position using an incremental Bayesian estimation (step 2a in (**a**)) and subsequently predict the upcoming grid location based on that information (step 3a+ in (**a**)). When the tiger-roar is *not* observed from the tiger door predicted based on information from earlier trials ($s_{TD,t}$), the grid location is re-estimated using the current observation v_t and the probabilities at trial t', which is the number of first trials after transferring to the current grid, as the prior distribution (step 3a- in (**a**)). The numbers assigned to each block correspond to the processing steps described in the **Methods**, in particular the **Behavioural model**, which also includes details of the top-down inference model (**b**) and the parallel inference model (**c**).

Supplementary Fig. 3: Behavioural analysis results compared between the hierarchical inference model and the alternative inference models.

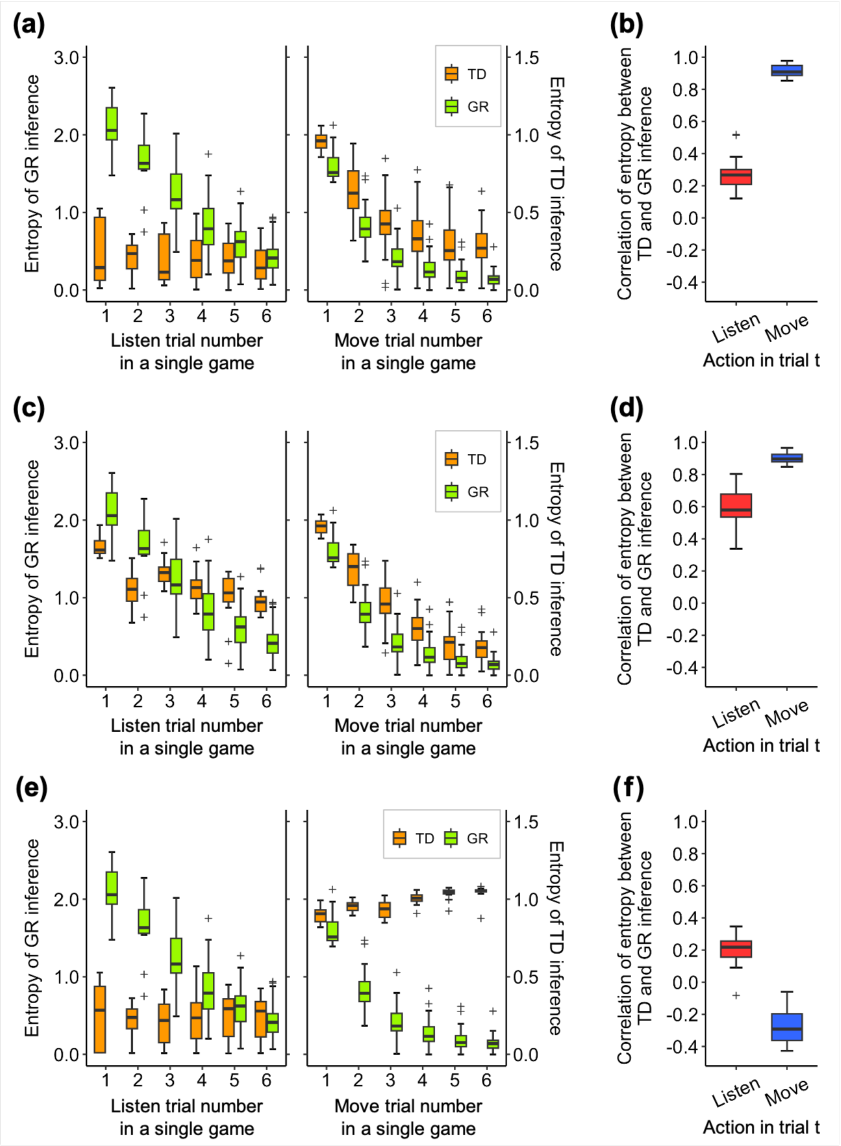

(a, b), **(c, d)**, and **(e, f)** show the results of model-based behavioural analysis using the hierarchical, top-down, and parallel inference models, respectively. The analysis methods are the same as those shown in Fig. 3b and e.

Supplementary Fig. 4: Brain activation involved in processing the listening action feedback.

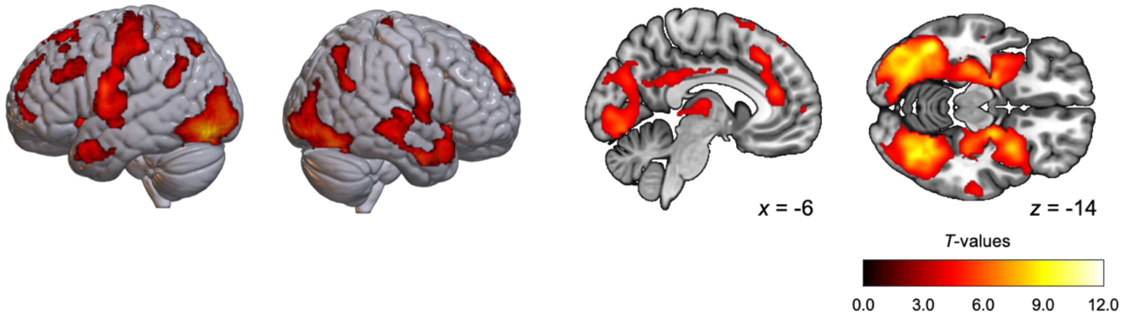

Brain regions that exhibited greater levels of activity at the onset of the action feedback in the Listen trials than in the Move trials. The clusters are significant at $p<0.05$ FWE-corrected, with the cluster-defining threshold $p<0.001$ uncorrected.

Supplementary Tables

Supplementary Table 1: Peak voxels of the regions that showed significantly higher activity in the action feedback period after the Listen than after the Move trials.

Region of activation	L/R	MNI coordinates			z-value	No. of voxels
Inferior temporal lobule	L/R	-44	-54	-12	6.15	30025
Superior frontal lobule	R	22	56	36	5.01	701
Supramarginal gyrus	L	-50	-50	26	4.66	409
Superior frontal gyrus	L	-28	58	2	4.61	384
Dorsal anterior cingulate cortex	L	-8	34	18	4.56	1481
Angular gyrus	R	58	-58	26	4.33	640
Precentral gyrus	R	22	-30	70	3.96	282

Supplementary Table 2: Peak voxels of the regions that showed activation in the re-estimation or updating trials during the action feedback period after the Listen action.

Region of activation	L/R	MNI coordinates			z-value	No. of voxels	p value (voxel level; FWE-corrected)
**Re-estimate mode > Updating mode**							
Insula	R	28	26	-4	4.24	302	0.038
Insula	L	-28	22	-12	4.36	194	0.128
Dorsomedial prefrontal cortex	L	6	40	44	4.04	193	0.129
**Updating mode > Re-estimation mode**							
Supplementary motor area	L	-8	-8	62	5.03	1502	2.87×10^{-6}
Fusiform gyrus	L	-48	-22	26	3.77	284	0.046

Supplementary Table 3: Peak voxels in areas where evoked activity was negatively correlated with two prediction confidence levels during the corresponding prediction period.

Region of activation	L/R	MNI coordinates			z-value	No. of voxels
**Tiger door prediction confidence**						
Supplementary motor area	L	-6	14	54	4.45	1132
Medial frontal gyrus	L	-38	14	30	4.20	802
Inferior parietal lobule	L	-38	-40	44	3.91	364
**Grid prediction confidence**						
Middle occipital gyrus	L	-46	-64	-8	4.67	600
Middle temporal gyrus	R	42	-76	20	4.31	857
Superior parietal lobule	R	30	-54	54	4.24	655
Insula	L	-28	18	4	4.17	230
Precuneus	L	-20	-70	32	4.15	1204
Inferior temporal gyrus	R	42	-64	-10	3.94	319

Supplementary Table 4: Brain areas where evoked activity was positively correlated with the entropy of the tiger's door inference and the grid inference, respectively, during the corresponding prediction period, and the statistics. The MNI coordinates indicate the peak voxel in each area.

Region of activation	L/R	MNI coordinates			z-value	No. of voxels
**Entropy of tiger door inference**						
Dorsomedial prefrontal cortex	L	-6	6	60	5.60	6693
Inferior frontal gyrus	R	34	22	18	4.55	2486
Inferior parietal lobule	L	-42	-34	40	4.48	362
Superior temporal gyrus	R	50	-24	-2	4.43	1279

Middle temporal gyrus	L	-54	-52	-2	4.37	500
Inferior parietal lobule	R	32	-44	44	3.83	311
**Entropy of grid inference**						
Fusiform gyrus	L	-42	-66	-14	5.48	1164
Calcarine sulcus	L	-18	-68	14	5.42	3044
Middle temporal gyrus	R	44	-76	16	4.93	2676
Lateral geniculate nucleus	L	-24	-22	-4	4.84	272
Putamen	R	24	14	-2	4.78	616
Putamen	L	-20	18	-2	4.68	870
Inferior frontal gyrus	R	36	6	26	4.60	303
Dorsomedial prefrontal cortex	L	-8	20	44	4.38	1226
Parahippocampal gyrus	R	22	-40	-4	4.28	1274
Superior frontal gyrus	R	26	6	56	4.14	589
Precentral gyrus	L	-46	2	30	3.76	227

Supplementary Table 5: The amount of reward for the prediction of the tiger door position and grid location. More points are awarded for higher confidence if the prediction is correct, and more points are deducted for higher confidence if the prediction is incorrect.

	**Tiger door prediction**		**Grid prediction**	
	High conf	Low conf	High conf	Low conf
Correct	1	1/2	2	1/3
Incorrect	−1	−1/2	−2	−1/12
