# Supplementary material for: Belief inference for hierarchical hidden states in spatial navigation
Source: Commun Biol. 2024 May 21;7:614. doi: 10.1038/s42003-024-06316-0 (PMC11109253; doi:10.1038/s42003-024-06316-0)
Supplement: Supplementary file 3 — Reporting summary [file 42003_2024_6316_MOESM3_ESM.pdf]

nature portfolio

Corresponding author(s): Risa Katayama

Last updated by author(s): Apr 19, 2024

Reporting Summary

Nature Portfolio wishes to improve the reproducibility of the work that we publish. This form provides structure for consistency and transparency in reporting. For further information on Nature Portfolio policies, see our Editorial Policies and the Editorial Policy Checklist.

Statistics

For all statistical analyses, confirm that the following items are present in the figure legend, table legend, main text, or Methods section.

n/a	Confirmed	
☐	☒	The exact sample size (n) for each experimental group/condition, given as a discrete number and unit of measurement
☐	☒	A statement on whether measurements were taken from distinct samples or whether the same sample was measured repeatedly
☐	☒	The statistical test(s) used AND whether they are one- or two-sided<br>*Only common tests should be described solely by name; describe more complex techniques in the Methods section.*
☒	☐	A description of all covariates tested
☐	☒	A description of any assumptions or corrections, such as tests of normality and adjustment for multiple comparisons
☐	☒	A full description of the statistical parameters including central tendency (e.g. means) or other basic estimates (e.g. regression coefficient) AND variation (e.g. standard deviation) or associated estimates of uncertainty (e.g. confidence intervals)
☐	☒	For null hypothesis testing, the test statistic (e.g. F, t, r) with confidence intervals, effect sizes, degrees of freedom and P value noted<br>*Give P values as exact values whenever suitable.*
☒	☐	For Bayesian analysis, information on the choice of priors and Markov chain Monte Carlo settings
☒	☐	For hierarchical and complex designs, identification of the appropriate level for tests and full reporting of outcomes
☐	☒	Estimates of effect sizes (e.g. Cohen's d, Pearson's r), indicating how they were calculated

Our web collection on statistics for biologists contains articles on many of the points above.

Software and code

Policy information about availability of computer code

Data collection	Behavioral data: Psychopy3 (Peirce et al., 2019).<br>MRI data: 3T Siemens MAGNETOM Prisma fit scanner.
Data analysis	Behavioral analyses: Matlab R2023a and R 4.2.3.<br>Imaging analyses: SPM12 (Wellcome Department of Cognitive Neurology, London, UK).

For manuscripts utilizing custom algorithms or software that are central to the research but not yet described in published literature, software must be made available to editors and reviewers. We strongly encourage code deposition in a community repository (e.g. GitHub). See the Nature Portfolio guidelines for submitting code & software for further information.

Data

Policy information about availability of data

All manuscripts must include a data availability statement. This statement should provide the following information, where applicable:

- Accession codes, unique identifiers, or web links for publicly available datasets
- A description of any restrictions on data availability
- For clinical datasets or third party data, please ensure that the statement adheres to our policy

Anonymized behavioural data are available on GitHub (https://github.com/RisaKatayama/article-belief-hierarchical-spatial.git). Unthresholded group-level statistical maps are available on NeuroVault (https://neurovault.org/collections/NZJMDMFQ/).

Research involving human participants, their data, or biological material

Policy information about studies with human participants or human data. See also policy information about sex, gender (identity/presentation), and sexual orientation and race, ethnicity and racism.

Reporting on sex and gender	20 participants (14 males and 6 females; sex was determined by self-report). Because sex is not a relevant factor within our study, this information was reported only for purposes of characterising our participants.
Reporting on race, ethnicity, or other socially relevant groupings	N/A
Population characteristics	Age range of the participants was 20-29 (22.1±1.9 [mean ± SD]). No other data regarding participants was collected for the analyses. All participants were recruited from he Kyoto University community.
Recruitment	Participants were recruited from the Kyoto University community through social networking service (LINE) advertising.
Ethics oversight	This study was approved by the ethics committees of the Advanced Telecommunications Research Institute International, Japan and the Graduate School of Informatics, Kyoto University, Japan.

Note that full information on the approval of the study protocol must also be provided in the manuscript.

Field-specific reporting

Please select the one below that is the best fit for your research. If you are not sure, read the appropriate sections before making your selection.

☒ Life sciences ☐ Behavioural & social sciences ☐ Ecological, evolutionary & environmental sciences

For a reference copy of the document with all sections, see nature.com/documents/nr-reporting-summary-flat.pdf

Life sciences study design

All studies must disclose on these points even when the disclosure is negative.

Sample size	The minimum number of participants was defined as 18, based on a power analysis (α=0.05, 1–β=0.8), with the effect size calculated from a previous study on hierarchical decision-making using G*Power (http://www.gpower.hhu. de/).
Data exclusions	Data from all of the participants included the analyses.
Replication	N/A
Randomization	N/A; only one group in the study
Blinding	N/A; only one group in the study

Reporting for specific materials, systems and methods

We require information from authors about some types of materials, experimental systems and methods used in many studies. Here, indicate whether each material, system or method listed is relevant to your study. If you are not sure if a list item applies to your research, read the appropriate section before selecting a response.

Materials & experimental systems

n/a	Involved in the study	
☒	☐	Antibodies
☒	☐	Eukaryotic cell lines
☒	☐	Palaeontology and archaeology
☒	☐	Animals and other organisms
☒	☐	Clinical data
☒	☐	Dual use research of concern
☒	☐	Plants

Methods

n/a	Involved in the study	
☒	☐	ChIP-seq
☒	☐	Flow cytometry
☐	☒	MRI-based neuroimaging

Plants

Seed stocks	*Report on the source of all seed stocks or other plant material used. If applicable, state the seed stock centre and catalogue number. If plant specimens were collected from the field, describe the collection location, date and sampling procedures.*
Novel plant genotypes	*Describe the methods by which all novel plant genotypes were produced. This includes those generated by transgenic approaches, gene editing, chemical/radiation-based mutagenesis and hybridization. For transgenic lines, describe the transformation method, the number of independent lines analyzed and the generation upon which experiments were performed. For gene-edited lines, describe the editor used, the endogenous sequence targeted for editing, the targeting guide RNA sequence (if applicable) and how the editor was applied.*
Authentication	*Describe any authentication procedures for each seed stock used or novel genotype generated. Describe any experiments used to assess the effect of a mutation and, where applicable, how potential secondary effects (e.g. second site T-DNA insertions, mosiacism, off-target gene editing) were examined.*

Magnetic resonance imaging

Experimental design

Design type	Task functional MRI; event-related design
Design specifications	Participants performed 24 games (237.2±22.0 trials) divided into 8 sessions; 4 sessions (12 games) in the behavioral experiment outside of the scanner, and another 4 session (12 games) in the MRI scanning experiment on the same day. Each game comprised 2–14 trials (10.9±2.4 trials per game).<br>Trial procedure was as follows: i) ITI (1.5s), ii) action choice (up to 4s), iii) action feedback (1.5s), iv) delay for the tiger door prediction (4s), v) choice for the tiger door prediction (up to 2s), vi) response feedback (1.5s), vii) delay for the tiger door prediction confidence (4s), viii) choice or the tiger door prediction confidence (up to 2s), ix) response feedback (1.5s), x) delay for the grid prediction (4s), xi) choice for the grid prediction (row) (up to 2s), xii) choice for the grid prediction (col) (up to 2s), xiii) response feedback (1.5s), xiv) delay for the grid prediction confidence (4s), xv) choice or the grid prediction confidence (up to 2s), ix) response feedback (1.5s).
Behavioral performance measures	We recorded the participants' actions, prediction of the tiger door position and the grid location in the maze, and the confidence levels (high or low) of these two types of prediction respectively. The behavioral performance, i.e., the prediction accuracy was 80.6±3.5% (tiger door prediction) and 57.3±8.6% (grid prediction), respectively, and both prediction accuracies were significantly higher than chance (Wilcoxon signed-rank test; tiger door, $p<0.0001$; grid, $p<0.0001$). The prediction accuracies were also higher when the participants reported a high confidence level than when they reported a low confidence level (tiger door, $p<0.0001$; grid, $p<0.0001$).

Acquisition

Imaging type(s)	functional and structual
Field strength	3T
Sequence & imaging parameters	interleaved T2*-weighted echo-planar images (TR, 1000 ms; TE, 30 ms; flip angle, 50°; matrix size, 100×100; field of view, 200×200; voxel size, 2×2×2.5 mm; and number of slices, 66) and a standard MPRAGE sequence (TR, 2250 ms; TE, 3.06 ms; flip angle, 9°; field of view, 256×256; voxel size, 1×1×1 mm).
Area of acquisition	whole brain

Diffusion MRI ☐ Used ☒ Not used

Preprocessing

Preprocessing software	SPM12 (slice-timing correction, spatial realignment, co-registration to the individual high-resolution anatomical image, normalisation to an MNI template, smoothing with a Gaussian kernel filter (FWHM, 8 mm)).
Normalization	Each participant's high-resolution T1-weighted structural image was coregistered to the individual mean functional image. Using the segmentation approach implemented in SPM12, forward deformation fields were obtained and then were used to normalize the functional images to MNI space.
Normalization template	MNI template included in SPM12 were used
Noise and artifact removal	Six motion correction parameters for each session produced during realignment were used as nuisance variables in generalised linear model (GLM) analyses. To remove low-frequency drifts, functional images were high-pass filtered with a cut-off of 128s.
Volume censoring	N/A

Statistical modeling & inference

Model type and settings: We used a mass-univariate GLM analysis on the first level. A random effects analysis was performed at the second level.

Effect(s) tested: A simple t-test in SPM12 was used to assess differences in the contrast between in the action feedback phase in the Listen trials and the Move trials and that between two independent modes of information processing (based on our behavioroal model) in the action feedback phase of the Listen trials, and to assess the effect of the prediction confidence and the entropy calculated from our model.

Specify type of analysis: ☒ Whole brain ☐ ROI-based ☐ Both

Statistic type for inference (See Eklund et al. 2016): Voxel-wise statistics were used.

Correction: FWE correction implemented in SPM12 (cluster level)

Models & analysis

n/a	Involved in the study
☒	☐ Functional and/or effective connectivity
☒	☐ Graph analysis
☒	☐ Multivariate modeling or predictive analysis
